# Supplementary material for: Seeing oneself as a data reuser: How subjectification activates the drivers of data reuse in science
Source: PLoS One. 2022 Aug 18;17(8):e0272153. doi: 10.1371/journal.pone.0272153 (PMC9387815; doi:10.1371/journal.pone.0272153)
Supplement: S1 File — (PDF) [file pone.0272153.s001.pdf]

Interview questions for researchers	Interview questions for intermediaries
For what purpose did you reuse / consider reusing the data?	For what purpose are you/your organization facilitating research data reuse?
Which data did you use / consider using?	Which data are you facilitating the reuse of?
Who did the data belong to?	Who does the data belong to?
Where did the data come from?	Where did the data come from?
We are particularly interested in the “mechanisms” of how data gets from the sharer to the reuser (e.g., how it can be found and extracted, what further processing is needed, etc.) Could you provide us with some information on this?	We are particularly interested in the “mechanisms” of how data gets from the sharer to the reuser (e.g., how it is found and extracted, what further processing is needed, etc.) Could you provide us with some information on this?
We are also interested in different aspects of timing as concerns data reuse. At what time did you reuse data<br>a. In your own project<br>b. In your career<br>c. As concerns the availability of data	We are also interested in different aspects of timing as concerns data reuse. As far as you can tell, at what time did you / your organisation facilitate data reuse?<br>a. In research projects<br>b. In the individual career of researchers<br>c. As concerns the availability of data
What helped and hindered you in your reuse of data?	In your experience as an intermediary, what helps and/or hinders in the reuse of data?
If you tried to reuse data but ultimately failed, why was this the case in your opinion?	As an intermediary, are you aware of cases where reuse of research data was attempted but ultimately failed? If so, why was this the case in your opinion?
On a scale from 1 (bad experience) to 10 (good experience), how would you rate your data reuse experience? Why?	On a scale from 1 (bad experience) to 10 (good experience) how would you rate the current data reuse experience? Why?
On a scale from 1 (not likely) to 10 (most likely) how likely would you be to reuse data in the future? Why (not)?	On a scale from 1 (not likely) to 10 (most likely) how likely is it that data reuse takes place in the future? Why (not)?
How can data reuse be promoted?	How can data reuse be promoted?
Are there any final thoughts you want to share concerning your data reuse experience?	Are there any final thoughts you want to share concerning data reuse?
