# Supplementary material for: Seeing oneself as a data reuser: How subjectification activates the drivers of data reuse in science
Source: PLoS One. 2022 Aug 18;17(8):e0272153. doi: 10.1371/journal.pone.0272153 (PMC9387815; doi:10.1371/journal.pone.0272153)
Supplement: S2 File — (PDF) [file pone.0272153.s002.pdf]

Written follow-up questions for researchers
Would you say that you see yourself as a data reuser?
Can you briefly explain why you (do not) see yourself as a data reuser?
When did you first come to believe that data reuse could be a useful part of your research and/or teaching practice?
Can you briefly describe the situation or context in which you formed this belief?
How many times have you subsequently reused data?
