# Supplementary material for: Seeing oneself as a data reuser: How subjectification activates the drivers of data reuse in science
Source: PLoS One. 2022 Aug 18;17(8):e0272153. doi: 10.1371/journal.pone.0272153 (PMC9387815; doi:10.1371/journal.pone.0272153)
Supplement: S3 File — (PDF) [file pone.0272153.s003.pdf]

	Activating mechanism for recurrring reuse	Project-independent factors influencing reuse behavior			Project-dependent factors influencing reuse behavior			
Participant	Subjectification	Researcher attitudes	Community norms	Rewards and requirements	Data characteristics	Trust in the data	Suitability for purpose	Capabilities to reuse
1		X			X	X	X	
2	X	X		X	X	X	X	X
3	X	X			X	X		X
4	X	X	X	X	X		X	X
5					X		X	
6			X		X	X		
7			X	X	X	X		
8			X	X	X			
9	X			X	X	X		
10	X		X		X	X		
11	X	X		X	X	X	X	X
12	X		X		X	X		X
13	X	X	X	X	X	X		X
14	X			X	X			
15				X	X	X		
16		X	X		X	X		
17	X				X			X
18				X	X	X		X
19				X	X	X		
20		X		X	X	X	X	
21	X				X	X		
22				X	X			
23	X	X	X	X	X	X	X	
24			X		X			X
