# Supplementary material for: Seeing oneself as a data reuser: How subjectification activates the drivers of data reuse in science
Source: PLoS One. 2022 Aug 18;17(8):e0272153. doi: 10.1371/journal.pone.0272153 (PMC9387815; doi:10.1371/journal.pone.0272153)
Supplement: Attachment — Submitted filename: Response to Reviewers.docx [file pone.0272153.s005.docx]

We would like to thank the reviewer for their additional comments and suggestions. Please see our point-by-point responses below.

**Reviewer #2 Comments**

*It's wonderful to see that authors have made some effective and clear changes*.

- Thank you for these encouraging comments; we are pleased to hear that our previous round of revisions clarified the intervention that we are seeking to make.

*The article takes the cross-level relationship between influencing factors as an important innovation of this paper, rather than the discovery of factors. Based on the review and participant descriptions, I assume that this cross-level relationship is one in which a factor exerts its influence across both institutional and individual levels, correct? If so, please describe it clearly. If not, please also add what levels it is across. The current description makes it easy to assume that a factor is acting across both project-independent and project-independent dimensions (although the authors clearly state this in their reply comments).*

- Thank you for pointing to the ambiguity in our use of the term “cross-level relationship.” To resolve this, we have introduced a distinction between factor type and level of analysis. Each of the seven influencing factors is associated with one of two factor types: project-independent or project-dependent. Each of the seven factors is also associated with one of three levels of analysis: individual, project, or institutional level. We have updated our model (Fig 1) to reflect this distinction, and have specified when we are referring to factor type vs. level of analysis throughout the manuscript.

*Furthermore, do all factors have cross-level relationships? Or only some of them? What is the role played by the same factor across levels? The current results section mentions this situation and expresses this relationship in a rather fragmented and implicit formulation, which needs to be explored in depth in the discussion.*

- Thank you for these insightful questions, which helped us to recognize not only some linguistic ambiguity but also conceptual imprecision in our use of the term “cross-level relationship.” For the sake of clarity, this term no longer appears in our manuscript. Our position is not that individual factors have cross-level relationships (although our data show that some project-level factors, including trust in the data and capabilities to reuse, are shaped by both individual and institutional conditions). Rather, our position is that all seven factors, taken together, exert an influence on recurring reuse behavior across levels of analysis, which we are able to explain by reference to the activating mechanism of subjectification as an inherently multilevel process. We have spelled out this argument explicitly in the revised second paragraph of the Discussion section (lines 764-783).
